# Regional Patterns of Multimorbidity and Hospitalization in Saskatchewan’s Aging Population

**DOI:** 10.3390/healthcare14020191

**Published:** 2026-01-12

**Authors:** Udoka Okpalauwaekwe, Masud Rana, Huey-Ming Tzeng

**Affiliations:** 1 Department of Family Medicine, College of Medicine, University of Saskatchewan, Saskatoon, SK S7N 2Z4, Canada; 2 School of Nursing, The University of Texas Medical Branch, Galveston, TX 77555, USA; tzenghm@gmail.com

**Keywords:** multimorbidity, older adults, Saskatchewan, hospitalization, emergency room use, health equity, chronic disease, regional variation, rural health, aging population

## Abstract

**Background**: Multimorbidity (the co-occurrence of two or more chronic conditions) is increasingly common among older adults and contributes to diminished well-being and greater healthcare use. While national data highlight regional variation, few studies have examined how multimorbidity is patterned within provinces like Saskatchewan or how it relates to access and acute care use. **Objective**: To describe sociodemographic and geographic patterns of multimorbidity among older adults in Saskatchewan and examine its association with healthcare access, unmet needs, and recent emergency department (ED) visits and hospitalizations. **Methods**: We conducted a secondary analysis of a population-based telephone survey of 1093 adults aged 65+ across Saskatchewan. Respondents were categorized by chronic disease burden (none, one, or multimorbidity). Descriptive statistics and postal code-level mapping explored health status, access, and utilization. **Results**: Multimorbidity (10.6%) was more prevalent among older adults aged 75+, Indigenous respondents, and those with lower education. It was associated with poorer self-rated health, greater unmet needs, and higher ED visits (20.7%) and hospitalizations (12.1%) compared to those without chronic conditions. Northern regions had proportionally higher multimorbidity, despite smaller populations. **Conclusions**: Findings highlight social and spatial disparities in chronic disease burden and underscore the need for equity-focused strategies in Saskatchewan’s rural and northern communities.

## 1. Introduction

Multimorbidity (commonly defined as the co-occurrence of two or more chronic conditions) is fast becoming a growing public health concern among older adults in Canada, with prevalence estimates exceeding 60% in seniors [1,2,3]. Unlike comorbidity, which denotes secondary conditions tied to a primary illness, multimorbidity captures the interwoven complexity of multiple chronic diseases that jointly impair functional ability, increase care needs, and erode quality of life [4,5]. Among older adults, this pattern is becoming increasingly normative. In Canada, the most prevalent multimorbidity combination among adults aged 45 and older is osteoarthritis and hypertension [6,7]. However, the specific conditions captured in multimorbidity studies often vary by context and data source. In Saskatchewan, regional health planning has emphasized other high-burden conditions such as diabetes, heart failure, and chronic respiratory diseases (particularly among older and rural populations) warranting focused investigation in these areas. As Geda et al. (2021) [4] notes, risk increases with age, female sex, retirement status, poor self-rated health, abnormal BMI, and sleep disturbances [6,7]. Socioeconomic disparities further influence risk: lower household income, material deprivation, and lower educational attainment are associated with higher multimorbidity prevalence and more frequent healthcare utilization [8,9,10,11].

Geographically, patterns of multimorbidity show significant regional and urban-rural variation in Canada, though findings differ by measurement approach and population studied. Some evidence suggests higher multimorbidity prevalence in urban cores compared to peripheral or rural communities, while other studies show mixed results, with rurality associated with both increased and decreased multimorbidity depending on context [12,13]. With regard to provincial variation, residents of Atlantic Canada, Ontario, and Quebec have shown increased odds of certain multimorbidity patterns (specifically osteoarthritis-hypertension), though at least one study found multimorbidity to be less likely among Quebec residents when examining overall multimorbidity prevalence [7,14]. However, area-level material deprivation consistently shows stronger associations with multimorbidity than geography alone, and multimorbidity rates in Ontario increased with material deprivation even after accounting for geographic variation [12,15,16].

Saskatchewan–a geographically diverse province with pronounced rural and Indigenous populations–has received relatively little focused attention in multimorbidity research specifically, despite its unique demographic characteristics [17,18]. Health system utilization varies by geography with rural residents showing higher emergency department use, while hospitalization rates are more strongly influenced by area-level deprivation than by rurality per se [19,20,21]. In Saskatchewan, provincial estimates suggest that 1 in 4 adults over age 65 live with three or more chronic conditions [22,23,24]. However, few studies have examined how this burden is distributed regionally or how it maps onto patterns of healthcare utilization especially in relation to hospitalizations and emergency room visits. A clearer understanding of intra-provincial disparities in multimorbidity can support more equitable health system responses, including improved chronic disease management, enhanced integration of services, and place-based resource allocation, which is why this study aims to: (1) describe the demographic and geographic distribution of multimorbidity among community-dwelling older adults in Saskatchewan; and (2) examine the association between chronic disease burden and self-reported acute care use (emergency department visits and hospitalizations). In visualizing and mapping out these patterns, we aim to inform more responsive and equitable approaches to aging, care delivery, and system planning in Saskatchewan and similar jurisdictions.

## 2. Methods

### 2.1. Study Design and Data Source

This study is a secondary analysis of data from a province-wide telephone survey conducted in Saskatchewan, Canada, between 25 April 2018, and 14 April 2019. The survey captured sociodemographic, health status, and service use data from community-dwelling adults aged 65 years and older residing across both rural and urban regions of the province.

In this analysis, we aimed to (a) examine patterns of multimorbidity among older adults by chronic disease burden, and (b) describe geographic variation in multimorbidity and recent acute care use using postal code-based regional mapping. Sampling and data collection were supported by the Canadian Hub for Applied and Social Research (CHASR) at the University of Saskatchewan [25].

### 2.2. Ethical Approval

The study received ethics approval from the University of Saskatchewan Research Ethics Board (Beh 18–67). All participants provided verbal informed consent before participating, in accordance with the Declaration of Helsinki.

### 2.3. Sampling Strategy and Recruitment

Recruitment and data collection were facilitated by the Canadian Hub for Applied and Social Research (CHASR) at the University of Saskatchewan–formerly known as the Social Sciences Research Laboratories (SSRL) [25]. CHASR is a nationally recognized research facility that supports faculty, staff, and students conducting social science research through access to cutting-edge infrastructure, methodological expertise, and community-engaged research supports [25].

We calculated an a priori target sample size of 1000. To recruit a representative sample of community-dwelling older adults in Saskatchewan, we employed computer-assisted telephone interviewing (CATI) using a randomized list of telephone numbers purchased from ASDE Survey Sampler™ (Gatineau, QC, Canada) [26], a widely used Canadian vendor that provides validated sampling frames for public opinion research. This sampling strategy followed Canadian Radio-television and Telecommunications Commission (CRTC) guidelines for random digit dialing (RDD) and included both landline and mobile numbers, with 25.8% mobile numbers to reflect Saskatchewan’s proportion of mobile-only households. Although 35,393 numbers were initially dialed, many were non-operational, business lines, or unanswered. Ultimately, 1093 participants aged 65 or older completed the survey. A detailed disposition report is available upon request from CHASR.

While geographical position was not directly linked to sampling eligibility, postal codes were collected from all consenting participants and used for mapping regional distribution. This approach enabled analysis of spatial variation while preserving anonymity. Although the sample was not weighted to provincial census distributions, the age, gender, and geographic composition of respondents closely resembled provincial patterns for older adults based on Statistics Canada census data. Limitations related to potential selection bias are discussed further in this paper.

### 2.4. Survey Instruments

Two instruments were administered during the telephone interviews (see Supplementary File S1):

1. The Patient Action Inventory for Self-Care (PAISC): this is a validated tool assessing 57 self-care behaviors across 11 domains (e.g., medication use, physical activity, emotional regulation) using dichotomous (yes/no) responses. It measures perceived importance, willingness, and ability to perform each behavior. This tool has demonstrated strong reliability and construct validity in older adult populations [27,28]. The PAISC provides a validated scoring approach using three parallel Yes/No dimensions (importance, desire, and ability) for each of its 57 items, producing scores ranging 0–57 (or proportional 0.00–1.00) [27,28]. For this present study, we did not apply these composite scoring methods or report outcomes from this instrument. Outcomes and analysis findings were reported from the tailored health assessment questionnaire.
2. A tailored health assessment questionnaire: adapted from a similar study conducted in the U.S. and modified for Saskatchewan’s healthcare context [29,30]. This instrument captured participant demographics (e.g., age, sex, ethnicity, education level, first three digits of postal code, immigration status), as well as health indicators including self-rated health, access to care, healthcare service utilization, transportation availability, and presence of chronic conditions. Participants were asked to indicate whether they had ever been diagnosed with any of the following six chronic conditions: asthma, chronic obstructive pulmonary disease (COPD), coronary artery disease (CAD), depression, diabetes, and heart failure. These were presented as closed-ended questions (Yes/No) with no free-text input. The list was selected in collaboration with provincial health partners and informed by a prior qualitative study with older adults in Saskatchewan [29]. For the purpose of this analysis, multimorbidity was defined as the presence of two or more of these conditions.

This current analysis, however, focused exclusively on variables drawn from the health assessment questionnaire.

### 2.5. Key Variables and Outcome Measures

At the time of data collection, we included six conditions which included: asthma, chronic obstructive pulmonary disease (COPD), coronary artery disease (CAD), depression, diabetes, and heart failure. These conditions were selected at the time of survey development based on provincial priorities and perceived relevance to Saskatchewan’s older adult population pooled from a qualitative study we had published at the time [29]. While not a diagnostic framework per se, this prior qualitative study [29] provided contextual insight that informed the survey’s chronic condition list. Although this list reflects conditions known to drive health system use and complexity, we acknowledge that it is not exhaustive and may underestimate the broader burden of multimorbidity.

### 2.6. Morbidity Grouping

To explore patterns of disease burden, healthcare access, and well-being across older adults in Saskatchewan, we categorized respondents into three mutually exclusive morbidity groups based on their self-reported chronic conditions: (1) no chronic condition, (2) one chronic condition, and (3) multimorbidity (two or more chronic conditions). This grouping strategy was selected a priori to enable clear descriptive comparisons across a spectrum of chronic disease burden. Including a “No Chronic Condition” group served as an important reference point, allowing us to contrast health outcomes and service use among those with no identified burden against those with escalating levels of multimorbidity. While we acknowledge that people without chronic disease are not “at risk” for the same conditions (e.g., the reviewer’s concern about biologic plausibility), they remain a crucial comparison group in public health and aging studies, especially in highlighting disparities in perceived health, service access, and unmet needs.

Key outcome variables include: (a) access to healthcare services; (b) transportation availability; (c) self-rated quality of life and health domains (physical, mental, emotional, spiritual); (d) emergency room visits and hospitalizations within the past three months; and (e) unmet healthcare needs.

### 2.7. Data Analysis

We conducted descriptive statistical analyses (frequencies, cross-tabulations) to characterize patterns of multimorbidity and related outcomes. Given the exploratory and participatory design of this study, we did not conduct inferential statistical tests. Our intent was to generate actionable insights for regional health planning and inform future hypothesis-driven research. This was intentionally designed as such in response to early stakeholder questions and provincial health authority priorities. Accordingly, we focused on identifying patterns of chronic disease burden and regional variation rather than conducting predictive or causal inference. Plans are underway to extend this work using inferential models in linked administrative datasets and with a co-developed Saskatchewan-specific deprivation index. All analyses were performed using IBM SPSS Statistics version 28.0 (IBM Corp., Armonk, NY, USA). Postal codes were used to map participants’ geographic location by Forward Sortation Area (FSA) for spatial analysis.

## 3. Results

### 3.1. Sample Characteristics

A total of 1093 community-dwelling older adults in Saskatchewan participated in the study (Table 1). Among them, 673 (61.6%) reported no chronic conditions, 304 (27.8%) had one chronic condition, and 116 (10.6%) had multimorbidity (defined as two or more chronic conditions). The majority of respondents were aged 65–74 years (58.7%) and female (71.3%). Most participants identified as White (89.4%), while 6.0% identified as Indigenous. About 30.1% had completed a bachelor’s degree or higher, while one in five had no formal post-secondary education. Individuals with multimorbidity were more likely to report lower educational attainment and income levels. See Table 1 for more description.

### 3.2. Self-Reported Chronic Conditions and Multimorbidity Patterns (Based on Six-Condition List)

As shown in Table 2, multimorbidity was more common among older participants (≥75 years), Indigenous respondents, and those with lower education. Among those with multimorbidity, the most frequently reported conditions included diabetes (54.3%), depression (76.7%), coronary artery disease (43.1%), heart failure (29.3%), asthma (32.8%), and COPD (32.8%). These often co-occurred in complex combinations, reflecting high-care needs. See Table 2 for more description.

### 3.3. Quality of Life and Self-Perceived Health

Table 3 illustrates a strong gradient between chronic disease burden and perceived health. Respondents with no chronic conditions reported high satisfaction with physical (92.4%), mental (83.7%), and emotional (94.9%) health. By contrast, among those with multimorbidity, satisfaction dropped to 78.4% (physical), 56.9% (mental), and 82.8% (emotional), with a higher proportion expressing dissatisfaction, particularly with mental health (41.4%). See Table 3 for more description

### 3.4. Access to Care and Unmet Needs

Most participants reported access to healthcare and transportation when needed, but disparities were evident by disease burden (Table 3). Only 46.6% of those with multimorbidity had access to all needed health services, compared to 54.5% in those without chronic conditions. Unmet healthcare needs were more than double in the multimorbidity group (10.3%) vs. the no-condition group (3.9%).

### 3.5. Acute Care Utilization

As shown in Table 4, recent ER visits and hospitalizations were substantially more common in those with multimorbidity. About 20.7% reported an ER visit in the past three months, and 12.1% reported hospitalization, compared to 7.6% and 4.0%, respectively, among those without chronic conditions.

### 3.6. Geographic Variation in Multimorbidity

**Figure 1:**
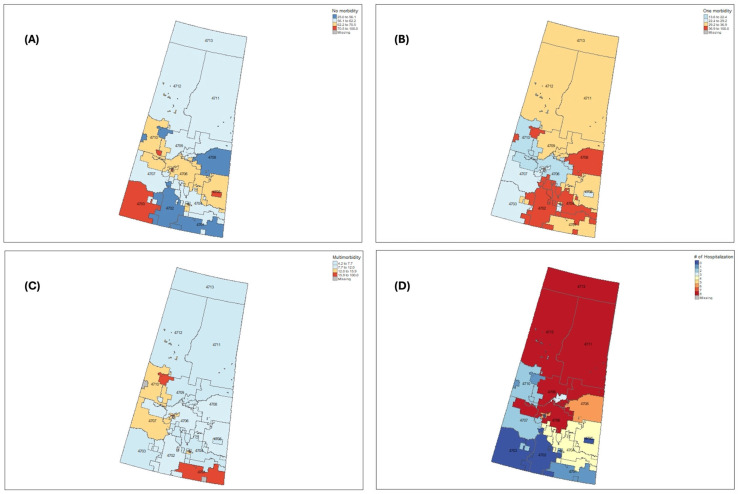
Geographic distribution of chronic disease burden and hospitalization among older adults in Saskatchewan. Panel (**A**): Proportion of respondents with no chronic condition. Panel (**B**): Proportion with one chronic condition. Panel (**C**): Proportion with multimorbidity (2+ conditions). Panel (**D**): Proportion reporting hospitalization in the past year. Values are based on self-reported data from 1093 older adults, aggregated at the Forward Sortation Area (FSA) level.

Regional mapping of multimorbidity patterns revealed striking geographic disparities across Saskatchewan (Figure 1). Although northern regions accounted for a smaller share of total survey respondents (Panel D), they had a disproportionately higher proportion of older adults reporting multimorbidity (Panel C). In contrast, southern and central regions (particularly urban-adjacent FSAs) had higher proportions of respondents with no chronic conditions (Panel A) or only one condition (Panel B). These patterns suggest that while overall population numbers are greater in the south, the relative burden of chronic disease may be more concentrated in northern and rural communities. This divergence between absolute volume and proportional burden underscores potential structural inequities in access to preventive care and supports the need for regionally tailored approaches to chronic disease management.

## 4. Discussion

This study offers one of the first regionally detailed, population-based snapshots of multimorbidity among older adults in Saskatchewan. While the overall prevalence of multimorbidity (10.6%) in our sample appears lower than national estimates, (which often exceed 60% in seniors depending on definitions and data sources) [31,32,33], the stratified patterns and spatial variations observed reflect deeper structural inequities and health system limitations within Saskatchewan. Rather than simply quantifying multimorbidity burden, our findings illuminate how that burden is unevenly distributed across sociodemographic and geographic lines, with implications for care access, quality of life, and acute care reliance.

Across our sample of 1093 older adults, over one-third reported at least one chronic condition, while 10.6% met our operational definition of multimorbidity (≥2 conditions). Among those with multimorbidity, a sizable proportion also reported unmet care needs, diminished well-being, and recent hospital use, highlighting a convergence of burden across domains. Importantly, these patterns were not isolated; they were consistently observed across rural and northern respondents, Indigenous participants, and individuals with lower educational attainment [29,34,35]. The prevalence of depression within this group (14.6%) also warrants attention given its implications for self-management, care coordination, and service satisfaction. These findings suggest that even within a relatively healthy-seeming older population, substantial heterogeneity in health burden and care experience exists, and can be surfaced through targeted descriptive analyses.

Consistent with the broader Canadian literature, older age, lower education, and Indigenous identity were associated with higher multimorbidity in our sample. These trends align with existing research showing that social and structural determinants (such as colonial legacies, cumulative disadvantage, and differential exposure to environmental stressors) shape chronic disease trajectories across the life course [12,14,16,31,36,37,38]. The relatively high proportion of Indigenous respondents reporting multimorbidity, while based on small numbers, echoes national disparities and underscores the need for Indigenous-governed, culturally safe models of chronic care and prevention [23,34,38]. Of note, our ongoing work co-developing a deprivation index with relevant stakeholders and *rightsholders* for Saskatchewan, drawing on census-linked material and social indicators, may offer future opportunities to more precisely quantify how area-level disadvantage intersects with individual-level chronic disease burden, especially in northern and rural regions.

A steep gradient in self-perceived health across morbidity strata is evident in our data. Compared to those without chronic conditions, individuals with multimorbidity reported significantly lower satisfaction across physical, mental, emotional, and spiritual domains. These findings are consistent with Canadian studies linking multimorbidity to diminished life satisfaction, functional impairment, and lower health confidence [5,11]. However, what often remains less visible is the compounding role of emotional and spiritual health dimensions frequently undervalued in standard measures yet clearly salient among older adults navigating complex care needs. This reaffirms the need for health systems to broaden how multimorbidity is understood and addressed, not merely as a tally of conditions, but as an embodied experience that disrupts wellbeing across multiple dimensions [23,34].

Despite overall high reported access to healthcare, respondents with multimorbidity were notably less likely to have access to all needed services and more likely to report unmet needs. While this finding is not new, it remains unresolved. Previous work shows that people with multiple chronic conditions often face fragmented care, overlapping appointments, contradictory treatment plans, and long wait times, especially when care systems remain oriented around single-disease pathways [18,39,40,41]. In Saskatchewan, where vast distances, primary care shortages, and limited specialist availability converge, these gaps are not merely administrative–they are structural [42,43]. Interestingly, even basic enablers such as transportation access (reported as near-universal across groups) may obscure qualitative differences in reliability, affordability, or cultural appropriateness. Future directions of this work would disaggregate these dimensions further, particularly in under-served regions. Transportation access was reported as near-universal across all morbidity groups (over 90%). However, this may obscure deeper barriers not captured in a binary access measure. In Saskatchewan, a province marked by vast distances, limited public transit, and harsh winter conditions, transportation plays a disproportionately large role in shaping access to timely, continuous care, particularly in rural and northern regions [44]. The presence of a vehicle or driver does not necessarily equate to reliable, affordable, or culturally safe transportation. Previous studies in Canada have found that lack of dependable transport can reduce adherence to regular primary care provider visits, limit access to preventive care, and exacerbate isolation among older adults in remote areas [42,44,45]. Our future directions will explore transportation quality and context-specific barriers in greater depth.

Our finding that one in five older adults with multimorbidity reported an emergency department (ED) visit in the past three months is notable and aligns with Canadian studies documenting elevated ED use among this population [46,47]. While our dataset does not capture the reasons for these visits, existing research highlights how such reliance on acute care can reflect systemic access challenges, particularly in settings with limited provider availability, after-hours options, or geographic barriers [2,42,48,49,50]. In this context, ED visits may be clinically appropriate in many cases; however, repeated reliance may also indicate upstream system limitations, such as gaps in timely primary care access [51], inadequate medication management [48], or caregiver burnout [46,49]. Rather than labeling these individuals as “frequent users,” our findings prompt a shift toward understanding the context in which emergency care becomes the default. In northern and rural communities, where after-hours services or walk-in clinics are often limited or nonexistent, EDs may serve as the only reliable point of contact with the health system [8,50]. n Saskatchewan specifically, this dynamic is compounded by vast geographic distances and uneven provider distribution. These findings underscore the urgency of expanding community-based, integrated care models—tailored not only to chronic disease profiles, but also to local geography, cultural contexts, and system capacity.

Our maps reveal a nuanced but important pattern: while the absolute number of older adults with multimorbidity is highest in more populated southern regions, the proportional burden is greatest in the north. This divergence between volume and vulnerability should be central to health system planning. Resource allocation based solely on headcounts risks under-serving high-need but sparsely populated regions. When paired with deprivation metrics and local knowledge, spatial analyses like ours can guide the development of more place-based, equity-sensitive strategies. The province’s new commitments to primary care reform and team-based care models must be informed by such insights if they are to be effective for older adults with complex health needs [42].

### 4.1. Strengths and Limitations

Our study adds important detail to the relatively underexplored landscape of multimorbidity in Saskatchewan. Its strengths include (a) a large, population-based sample capturing rural, urban, and northern voices; (b) the use of stratified analysis and mapping to expose patterns often masked in aggregate data; and (c) relevance for aging and health equity policy agendas across Canada’s diverse jurisdictions.

However, several limitations warrant attention. As a cross-sectional survey, the data do not capture changes over time. Secondly, all information was self-reported, raising the possibility of recall bias or underreporting. Additionally, our original Patient Action Inventory for Self-Care provides a validated scoring approach using three parallel Yes/No dimensions (for importance, desire, and ability) for each of its 57 items, producing scores ranging 0–57 (or proportional 0.00–1.00). As our present study did not apply these composite scoring methods and instead used selected items descriptively, we acknowledge this as a methodological limitation and have identified it as an area for further analytic development. Third, the list of chronic conditions may not reflect the full spectrum of multimorbidity, especially neurological, musculoskeletal, or culturally specific diagnoses. Participants were asked to respond to a predefined list of six chronic conditions using closed-ended (yes/no) questions; no free-text or open-ended condition reporting was collected. Our definition of multimorbidity was therefore operationalized using six chronic conditions captured in the original survey (rather than a validated or comprehensive multimorbidity index), which may underestimate true disease burden and exclude common conditions such as hypertension, osteoarthritis, and cognitive disorders (which may underestimate true disease burden and exclude common conditions such as hypertension, osteoarthritis, and cognitive disorders, particularly among older adults). This constraint limits comparability with national studies and may bias prevalence estimates downward. Additionally, the number of small cell sizes in some geographic regions limit generalizability. Finally, functional limitations, disease severity, or medication burden were not assessed. Additionally, our use of a “no chronic condition” category as a descriptive comparator group (based on absence of self-reported conditions from the predefined list) does not imply biological immunity or inability to develop chronic illness and may reflect reporting differences rather than true absence of disease.

### 4.2. Future Directions and Implications

Findings from this study highlight that the geography of aging is not neutral: place, power, and policy matter. Addressing multimorbidity in older adults (particularly those in northern, rural, or equity-deserving contexts) requires moving beyond reactive care toward longitudinal, integrated, and relationship-based approaches that centre the full spectrum of what it means to age well.

Our future research would consider linking self-reported data to administrative records to better understand care pathways and service utilization over time. We are working with and engaging older adults, including Indigenous communities in a qualitative study to shed light on lived experiences, resilience strategies, and culturally grounded approaches to wellness.

This work has also laid the foundation for co-developing a provincial deprivation index, combining these indicators with multimorbidity data to strengthen its predictive utility and support resource prioritization. We intend to use this to evaluate the uptake and outcomes of emerging rural chronic care models, home-based supports, and Indigenous-led health initiatives.

## 5. Conclusions

This study offers one of the first province-wide descriptive snapshots of multimorbidity among older adults in Saskatchewan, revealing how chronic disease burden is shaped not only by age, but by geography, social position, and structural inequity. While many older adults reported good health and access to care, those with multimorbidity, particularly in northern and socioeconomically marginalized communities, faced notable disparities in physical, emotional, and spiritual wellbeing, unmet care needs, and greater reliance on emergency services. By integrating self-reported survey data with geospatial analysis, our study highlights the importance of understanding multimorbidity not as a uniform phenomenon, but as one deeply influenced by place, identity, and system responsiveness. Our findings affirm that multimorbidity is not evenly distributed, and that structural and spatial inequities shape both experience and outcome; and underscore the need for equity-oriented chronic disease strategies that reflect regional vulnerability, local care capacity, and the lived experiences of older adults.

Our future work on this will be linking these findings to administrative data, deprivation indices, and community-led insights that could further strengthen efforts to build responsive, integrated care systems that support healthy aging across Saskatchewan.

## Figures and Tables

**Table 1:** Sociodemographic Characteristics of Older Adults Stratified by Chronic Disease Status.

Characteristic	Total (N = 1093)	No Chronic Condition (N = 673)	1 Chronic Condition (N = 304)	2+ Chronic Conditions (N = 116)
Age (years)				
65–74	642 (58.7%)	409 (60.8%)	163 (53.6%)	70 (60.3%)
75–84	345 (31.6%)	201 (29.9%)	107 (35.2%)	37 (31.9%)
85+	99 (9.1%)	58 (8.6%)	32 (10.5%)	9 (7.8%)
Missing	7 (0.6%)	5 (0.7%)	2 (0.7%)	0 (0.0%)
Gender				
Female	779 (71.3%)	488 (72.5%)	209 (68.8%)	82 (70.7%)
Male	314 (28.7%)	185 (27.5%)	95 (31.2%)	34 (29.3%)
Ethnicity				
White	977 (89.4%)	611 (90.8%)	267 (87.8%)	99 (85.3%)
Indigenous	66 (6.0%)	31 (4.6%)	22 (7.2%)	13 (11.2%)
Asian	6 (0.5%)	3 (0.4%)	2 (0.7%)	1 (0.9%)
Black	1 (0.1%)	1 (0.1%)	0 (0.0%)	0 (0.0%)
Other	43 (3.9%)	27 (4.0%)	13 (4.3%)	3 (2.6%)
Highest Education Completed				
Bachelor’s degree or higher	329 (30.1%)	229 (34.0%)	75 (24.7%)	25 (21.6%)
Associate/diploma	251 (23.0%)	150 (22.3%)	69 (22.7%)	32 (27.6%)
High school	308 (28.2%)	172 (25.6%)	95 (31.2%)	41 (35.3%)
Some education	124 (11.3%)	74 (11.0%)	37 (12.2%)	13 (11.2%)
Other	69 (6.3%)	38 (5.6%)	26 (8.6%)	5 (4.3%)
Missing	12 (1.1%)	10 (1.5%)	2 (0.7%)	0 (0.0%)
Language				
English as first language	950 (86.9%)	586 (87.1%)	261 (85.9%)	103 (88.8%)
Not English	139 (12.7%)	83 (12.3%)	43 (14.1%)	13 (11.2%)
Missing	4 (0.4%)	4 (0.6%)	0 (0.0%)	0 (0.0%)

Key: Descriptive characteristics of older adults aged 65+ in Saskatchewan, stratified by the number of self-reported chronic conditions. Multimorbidity was defined as 2 or more chronic conditions. Note: Values are presented as count (percentage). Percentages may not total 100% due to rounding. “Missing” reflects unreported responses or ‘prefer not to say’.

**Table 2:** Self-Reported Chronic Conditions Among Older Adults by Morbidity Group (Based on a Predefined Six-Condition List).

Chronic Condition	Total (N = 1093)	1 Chronic Condition (N = 304)	2+ Chronic Conditions (N = 116)
Asthma	90 (8.2%)	52 (17.1%)	38 (32.8%)
Chronic Obstructive Pulmonary Disease (COPD)	51 (4.7%)	13 (4.3%)	38 (32.8%)
Coronary Artery Disease (CAD)	77 (7.0%)	27 (8.9%)	50 (43.1%)
Depression	145 (13.3%)	56 (18.4%)	89 (76.7%)
Diabetes	102 (9.3%)	39 (12.8%)	63 (54.3%)
Heart Failure	38 (3.5%)	4 (1.3%)	34 (29.3%)

Key: Self-reported prevalence of six common chronic conditions across older adult respondents in Saskatchewan, stratified by number of chronic diseases. Note: A total of 673 respondents did not report any of the six queried chronic conditions (asthma, COPD, CAD, depression, diabetes, or heart failure), and were excluded from condition-specific tabulations.

**Table 3:** Healthcare Access and Quality of Life Indicators Among Older Adults, Stratified by Chronic Disease Status.

Variable	Total (N = 1093)	No Chronic Condition (N = 673)	1 Chronic Condition (N = 304)	2+ Chronic Conditions (N = 116)
Access to healthcare when needed				
Yes (all services)	572 (52.3%)	367 (54.5%)	151 (49.7%)	54 (46.6%)
Yes (some services)	410 (37.5%)	242 (36.0%)	123 (40.5%)	45 (38.8%)
No	64 (5.9%)	39 (5.8%)	16 (5.3%)	9 (7.8%)
Missing	47 (4.3%)	25 (3.7%)	14 (4.6%)	8 (6.9%)
Transportation available when needed				
Yes	1050 (96.1%)	645 (95.8%)	294 (96.7%)	111 (95.7%)
No	32 (2.9%)	19 (2.8%)	9 (3.0%)	4 (3.4%)
Missing	11 (1.0%)	9 (1.3%)	1 (0.3%)	1 (0.9%)
Able to meet healthcare needs (past 3 months)				
Yes	1030 (94.2%)	641 (95.2%)	285 (93.8%)	104 (89.7%)
No	56 (5.1%)	26 (3.9%)	18 (5.9%)	12 (10.3%)
Missing	7 (0.6%)	6 (0.9%)	1 (0.3%)	0 (0.0%)
Quality of life (self-rated)				
Satisfied	1050 (96.1%)	645 (95.8%)	294 (96.7%)	111 (95.7%)
Not satisfied	32 (2.9%)	19 (2.8%)	9 (3.0%)	4 (3.4%)
Missing	11 (1.0%)	9 (1.3%)	1 (0.3%)	1 (0.9%)
Satisfaction with physical health				
Satisfied	972 (88.9%)	622 (92.4%)	259 (85.2%)	91 (78.4%)
Not satisfied	105 (9.6%)	43 (6.4%)	37 (12.2%)	25 (21.6%)
Missing	16 (1.5%)	8 (1.2%)	8 (2.6%)	0 (0.0%)
Satisfaction with mental health				
Satisfied	838 (76.7%)	563 (83.7%)	209 (68.8%)	66 (56.9%)
Not satisfied	238 (21.8%)	100 (14.9%)	90 (29.6%)	48 (41.4%)
Missing	17 (1.6%)	10 (1.5%)	5 (1.6%)	2 (1.7%)
Satisfaction with emotional health				
Satisfied	1010 (92.4%)	639 (94.9%)	275 (90.5%)	96 (82.8%)
Not satisfied	68 (6.2%)	24 (3.6%)	25 (8.2%)	19 (16.4%)
Missing	15 (1.4%)	10 (1.5%)	4 (1.3%)	1 (0.9%)
Satisfaction with spiritual health				
Satisfied	989 (90.5%)	625 (92.9%)	266 (87.5%)	98 (84.5%)
Not satisfied	83 (7.6%)	33 (4.9%)	34 (11.2%)	16 (13.8%)
Missing	21 (1.9%)	15 (2.2%)	4 (1.3%)	2 (1.7%)

Key: Healthcare access, service availability, and self-rated quality of life and well-being among older adults, stratified by number of self-reported chronic conditions. Note: Values are presented as count (percentage). “Satisfied” includes all positive self-ratings; “Not satisfied” includes negative or neutral ratings. Percentages may not total 100% due to rounding. “Missing” indicates unreported values.

**Table 4:** Health Service Utilization Among Older Adults, Stratified by Chronic Disease Status.

Variable	Total (N = 1093)	No Chronic Condition (N = 673)	1 Chronic Condition (N = 304)	2+ Chronic Conditions (N = 116)
Emergency Room Visit (past 3 months)				
Yes	116 (10.6%)	51 (7.6%)	41 (13.5%)	24 (20.7%)
No	974 (89.1%)	619 (92.0%)	263 (86.5%)	92 (79.3%)
Missing	3 (0.3%)	3 (0.4%)	0 (0.0%)	0 (0.0%)
Hospitalization (past 3 months)				
Yes	67 (6.1%)	27 (4.0%)	26 (8.6%)	14 (12.1%)
No	1023 (93.6%)	643 (95.5%)	278 (91.4%)	102 (87.9%)
Missing	3 (0.3%)	3 (0.4%)	0 (0.0%)	0 (0.0%)
Unmet Healthcare Needs (past 3 months)				
Yes	56 (5.1%)	26 (3.9%)	18 (5.9%)	12 (10.3%)
No	1030 (94.2%)	641 (95.2%)	285 (93.8%)	104 (89.7%)
Missing	7 (0.6%)	6 (0.9%)	1 (0.3%)	0 (0.0%)

Key: Self-reported emergency department visits, hospitalizations, and unmet healthcare needs among older adults in Saskatchewan, stratified by number of chronic conditions. Note: Values presented as count (percentage). “Missing” indicates respondents who did not provide a response to the relevant question. Percentages may not total 100% due to rounding.

## Data Availability

The raw data supporting the conclusions of this article will be made available by the authors on request.

## Supplementary Materials

The following supporting information can be downloaded at: https://www.mdpi.com/article/10.3390/healthcare14020191/s1, File S1: Patient Health Involvement Survey.
