# Supplementary material for: Regional Patterns of Multimorbidity and Hospitalization in Saskatchewan’s Aging Population
Source: Healthcare (Basel). 2026 Jan 12;14(2):191. doi: 10.3390/healthcare14020191 (PMC12841241; doi:10.3390/healthcare14020191)
Supplement: Supplementary file 1 [file healthcare-14-00191-s001.zip › healthcare-4057476 - Supplementary.pdf]

Patient Health Involvement Survey

INTRO1/INTRO3
Hello, my name is (FIRST NAME ONLY) and I am a student calling on behalf of Dr. Huey-Ming Tzeng from college of nursing at the University of Saskatchewan. We are conducting a 30-minute survey about health and self-care among older adults in Saskatchewan.

INTRO2.
Is there anyone in your household who is 65 years or older?
(IF YES) May I please speak to them?

1. Yes, speaking ***CONTINUE***
2. Yes, I'll get him/her ***REPEAT INTRODUCTION AND CONTINUE***
3. Not available ***ARRANGE CALLBACK***
4. *No one 65 years or older* ***END CALL***

INTRO4.
I would like to invite you to participate in this short survey. In this survey, we want to understand the things that you do when looking for or using health care. Participation is voluntary, and you have the right to withdraw at any time during the survey. The answers may either be for yourself or for a person you are providing care for. Your answers are important and will help the researcher to develop special programs for senior adults who need health care. Should you choose to withdraw at any point during the study, we will destroy any data collected beyond recovery. You can also skip questions you don't want to answer. This call will be recorded for quality control purposes. The information we collect is kept strictly confidential and none of the answers that you provide will be linked back to you personally. There are no known risks to participating in this survey. This research project has been approved on ethical grounds by the University of Saskatchewan Research Ethics Board. Any questions regarding your rights as a participant may be addressed to that committee through the Research Ethics Office at ethics.office@usask.ca (306) 966-2975 or toll free (888) 966-2975. If you have any questions about the study itself, you may contact Dr. Tzeng at 306-966-5568.

Are you willing to participate?

1. Yes ***CONTINUE***
2. No ***THANK AND END INTERVIEW***
3. Later/Not right now ***ARRANGE CALLBACK***

INTRO5.
Before we begin, may I please have your postal code?

IF RESPONDENT IS RELUCTANT, YOU CAN ASSURE THEM THAT THEIR POSTAL CODE WILL BE USED FOR STATISTICAL PURPOSES ONLY (TO UNDERSTAND DIFFERENCES BY REGION/GEOGRAPHY) AND WILL NOT BE USED TO IDENTIFY THEM IN ANY WAY.

ENSURE RESPONDENT PROVIDES COMPLETE SIX CHARACTER POSTAL CODE IN PROPER FORMAT (EXAMPLE : S7N 5A5).

THE FIRST BOX WILL ONLY ACCEPT AN 'S' (SK).

ENTER RESPONDENTS COMPLETE POSTAL CODE CHARACTERS:

1. (RECORD POSTAL CODE)

INTRO6.
(DO NOT READ)
RECORD SEX FROM RESPONDENT VOICE.

1. Male
2. Female

Part A. Patient Action Inventory for Self-Care

INTROA.
I am now going to ask you some questions about your self-care. Please answer Yes or No as to whether each of the following is important to you, whether you want to do it, and whether you are able to do it. You are free to answer only the questions that you are comfortable with.

**Behaviors**	A. Is this **important** to you?	B. Do you **want** to do this?	C. Are you **able** to do this?
A1. Find a doctor or practitioner who meets your needs	[ ] Yes<br>[ ] No	[ ] Yes<br>[ ] No	[ ] Yes<br>[ ] No
A2. Use available information to choose a doctor or practitioner	[ ] Yes<br>[ ] No	[ ] Yes<br>[ ] No	[ ] Yes<br>[ ] No
A3. Use data to choose a hospital or clinic	[ ] Yes<br>[ ] No	[ ] Yes<br>[ ] No	[ ] Yes<br>[ ] No
A4. Find a healthcare professional or group who agrees to see you over time	[ ] Yes<br>[ ] No	[ ] Yes<br>[ ] No	[ ] Yes<br>[ ] No
A5. Make a list of questions and issues to discuss at your appointment	[ ] Yes<br>[ ] No	[ ] Yes<br>[ ] No	[ ] Yes<br>[ ] No
A6. List your medications and take your list to appointments	[ ] Yes<br>[ ] No	[ ] Yes<br>[ ] No	[ ] Yes<br>[ ] No
A7. Be ready to talk about your medications and what they do	[ ] Yes<br>[ ] No	[ ] Yes<br>[ ] No	[ ] Yes<br>[ ] No
A8. Share all of your physical symptoms and history with your health care providers	[ ] Yes<br>[ ] No	[ ] Yes<br>[ ] No	[ ] Yes<br>[ ] No
A9. Share all your mental symptoms and history with your health care providers	[ ] Yes<br>[ ] No	[ ] Yes<br>[ ] No	[ ] Yes<br>[ ] No
A10. Ask your provider questions when needed	[ ] Yes<br>[ ] No	[ ] Yes<br>[ ] No	[ ] Yes<br>[ ] No
A11. Share your ideas about your care experience	[ ] Yes<br>[ ] No	[ ] Yes<br>[ ] No	[ ] Yes<br>[ ] No
A12. Make appointments, know the clinic's no show policy, and arrive on time	[ ] Yes<br>[ ] No	[ ] Yes<br>[ ] No	[ ] Yes<br>[ ] No
A13. Ask for special assistance when needed	[ ] Yes<br>[ ] No	[ ] Yes<br>[ ] No	[ ] Yes<br>[ ] No
A14. Bring your health insurance documents with you when you seek care	[ ] Yes<br>[ ] No	[ ] Yes<br>[ ] No	[ ] Yes<br>[ ] No
A15. Bring someone to help you move around when needed	[ ] Yes<br>[ ] No	[ ] Yes<br>[ ] No	[ ] Yes<br>[ ] No

A16. Bring someone with you to explain and take notes if needed	[ ] Yes<br>[ ] No	[ ] Yes<br>[ ] No	[ ] Yes<br>[ ] No
**Behaviors**	Is this **important** to you?	Do you **want** to do this?	Are you **able** to do this?
A17. Bring your recent test results and medical record when you seek care	[ ] Yes<br>[ ] No	[ ] Yes<br>[ ] No	[ ] Yes<br>[ ] No
A18. Ask your providers to share your medical record with each other	[ ] Yes<br>[ ] No	[ ] Yes<br>[ ] No	[ ] Yes<br>[ ] No
A19. Obtain records of your test results and clinic visits	[ ] Yes<br>[ ] No	[ ] Yes<br>[ ] No	[ ] Yes<br>[ ] No
A20. Find insurance that best matches you and your needs	[ ] Yes<br>[ ] No	[ ] Yes<br>[ ] No	[ ] Yes<br>[ ] No
A21. Apply for health insurance or social services when needed	[ ] Yes<br>[ ] No	[ ] Yes<br>[ ] No	[ ] Yes<br>[ ] No
A22. Know the payment limits of your insurance	[ ] Yes<br>[ ] No	[ ] Yes<br>[ ] No	[ ] Yes<br>[ ] No
A23. Change health insurance coverage as needed	[ ] Yes<br>[ ] No	[ ] Yes<br>[ ] No	[ ] Yes<br>[ ] No
A24. Keep recent health care receipts	[ ] Yes<br>[ ] No	[ ] Yes<br>[ ] No	[ ] Yes<br>[ ] No
A25. Seek more than one expert opinion for treatment of illness when needed	[ ] Yes<br>[ ] No	[ ] Yes<br>[ ] No	[ ] Yes<br>[ ] No
A26. Ask about the good and bad outcomes of suggested treatments	[ ] Yes<br>[ ] No	[ ] Yes<br>[ ] No	[ ] Yes<br>[ ] No
A27. Work with your health care provider(s) on your treatment plan	[ ] Yes<br>[ ] No	[ ] Yes<br>[ ] No	[ ] Yes<br>[ ] No
A28. Know side effects before starting new treatments	[ ] Yes<br>[ ] No	[ ] Yes<br>[ ] No	[ ] Yes<br>[ ] No
A29. Know of any interactions with your old and new treatments	[ ] Yes<br>[ ] No	[ ] Yes<br>[ ] No	[ ] Yes<br>[ ] No
A30. Fill or refill prescriptions on time	[ ] Yes<br>[ ] No	[ ] Yes<br>[ ] No	[ ] Yes<br>[ ] No
A31. Keep track of the results of your treatments	[ ] Yes<br>[ ] No	[ ] Yes<br>[ ] No	[ ] Yes<br>[ ] No
A32. Talk with your health care provider(s) when stopping your treatment	[ ] Yes<br>[ ] No	[ ] Yes<br>[ ] No	[ ] Yes<br>[ ] No
A33. Maintain all of your health care devices	[ ] Yes<br>[ ] No	[ ] Yes<br>[ ] No	[ ] Yes<br>[ ] No
A34. Discuss why tests are ordered before getting them done	[ ] Yes<br>[ ] No	[ ] Yes<br>[ ] No	[ ] Yes<br>[ ] No
A35. Track your symptoms and health measures	[ ] Yes<br>[ ] No	[ ] Yes<br>[ ] No	[ ] Yes<br>[ ] No
A36. Create habits that will improve health and prevent disease	[ ] Yes<br>[ ] No	[ ] Yes<br>[ ] No	[ ] Yes<br>[ ] No
A37. Find and use services that support your health behaviors	[ ] Yes<br>[ ] No	[ ] Yes<br>[ ] No	[ ] Yes<br>[ ] No
A38. Keep your new health behaviors going	[ ] Yes<br>[ ] No	[ ] Yes<br>[ ] No	[ ] Yes<br>[ ] No
A39. Follow the agreed treatment plan to	[ ] Yes	[ ] Yes	[ ] Yes

manage your symptoms	[ ] No	[ ] No	[ ] No
**Behaviors**	Is this **important** to you?	Do you **want** to do this?	Are you **able** to do this?
A40. Discuss use of health screening tests with your providers	[ ] Yes<br>[ ] No	[ ] Yes<br>[ ] No	[ ] Yes<br>[ ] No
A41. Seek early detection of diseases, for example: cancer	[ ] Yes<br>[ ] No	[ ] Yes<br>[ ] No	[ ] Yes<br>[ ] No
A42. Follow up on health screening results	[ ] Yes<br>[ ] No	[ ] Yes<br>[ ] No	[ ] Yes<br>[ ] No
A43. Get needed vaccines	[ ] Yes<br>[ ] No	[ ] Yes<br>[ ] No	[ ] Yes<br>[ ] No
A44. Participate in local health screening or wellness events	[ ] Yes<br>[ ] No	[ ] Yes<br>[ ] No	[ ] Yes<br>[ ] No
A45. Complete a living will and keep the file with your personal health records	[ ] Yes<br>[ ] No	[ ] Yes<br>[ ] No	[ ] Yes<br>[ ] No
A46. Assign a power of attorney and keep the file with your health records	[ ] Yes<br>[ ] No	[ ] Yes<br>[ ] No	[ ] Yes<br>[ ] No
A47. Talk to your family and health care provider about your living will	[ ] Yes<br>[ ] No	[ ] Yes<br>[ ] No	[ ] Yes<br>[ ] No
A48. Update and share your living will each year	[ ] Yes<br>[ ] No	[ ] Yes<br>[ ] No	[ ] Yes<br>[ ] No
A49. Seek to learn more about your own health and disease prevention	[ ] Yes<br>[ ] No	[ ] Yes<br>[ ] No	[ ] Yes<br>[ ] No
A50. Seek to learn about your chronic disease and your treatment options	[ ] Yes<br>[ ] No	[ ] Yes<br>[ ] No	[ ] Yes<br>[ ] No
A51. Know your personal health targets and what to do to meet them	[ ] Yes<br>[ ] No	[ ] Yes<br>[ ] No	[ ] Yes<br>[ ] No
A52. Use clinics for non-emergency care	[ ] Yes<br>[ ] No	[ ] Yes<br>[ ] No	[ ] Yes<br>[ ] No
A53. Stay free of tobacco products (e.g., cigarettes, cigars, chew)	[ ] Yes<br>[ ] No	[ ] Yes<br>[ ] No	[ ] Yes<br>[ ] No
A54. Seek to have meaningful social connections	[ ] Yes<br>[ ] No	[ ] Yes<br>[ ] No	[ ] Yes<br>[ ] No
A55. Limit your absence from your scheduled activities	[ ] Yes<br>[ ] No	[ ] Yes<br>[ ] No	[ ] Yes<br>[ ] No
A56. Choose to get involved in meaningful work	[ ] Yes<br>[ ] No	[ ] Yes<br>[ ] No	[ ] Yes<br>[ ] No
A57. Plan for the future	[ ] Yes<br>[ ] No	[ ] Yes<br>[ ] No	[ ] Yes<br>[ ] No

Part II. Patient Activation Measure

(Insignia Health. Patient Activation Measure; Copyright © 2003-2011. University of Oregon.)

I am now going to read you some statements that people sometimes make when they talk about their health. Please indicate whether you strongly disagree, disagree, agree, or strongly agree with each statement as it applies to you personally. Your answers should be what is true for you and not just what you think the doctor wants you to say.

B1.	When all is said and done, I am the person who is responsible for taking care of my health.	Disagree Strongly	Disagree	Agree	Agree Strongly	N/A
B2.	Taking an active role in my own health care is the most important thing that affects my health.	Disagree Strongly	Disagree	Agree	Agree Strongly	N/A
B3.	I know what each of my prescribed medications do.	Disagree Strongly	Disagree	Agree	Agree Strongly	N/A
B4.	I am confident that I can tell whether I need to go to the doctor or whether I can take care of a health problem myself.	Disagree Strongly	Disagree	Agree	Agree Strongly	N/A
B5.	I am confident that I can tell a doctor concerns I have even when he or she does not ask.	Disagree Strongly	Disagree	Agree	Agree Strongly	N/A
B6.	I am confident that I can follow through on medical treatments I may need to do at home.	Disagree Strongly	Disagree	Agree	Agree Strongly	N/A
B7.	I have been able to maintain (keep up with) lifestyle changes, like eating right or exercising.	Disagree Strongly	Disagree	Agree	Agree Strongly	N/A
B8.	I know how to prevent problems with my health.	Disagree Strongly	Disagree	Agree	Agree Strongly	N/A
B9.	I am confident I can figure out solutions when new problems arise with my health.	Disagree Strongly	Disagree	Agree	Agree Strongly	N/A
B10.	I am confident that I can maintain lifestyle changes, like eating right and exercising, even during times of stress.	Disagree Strongly	Disagree	Agree	Agree Strongly	N/A

Part III. The Positive and Negative Affect Schedule

(Granted permission to use this tool by the developer, Dr. Edmund R. Thompson, per correspondence dated January 29, 2015.)

On a scale of 1 to 5 where 1 is "Never" and 5 is "Always", thinking about yourself and how you normally feel, to what extent do you generally feel...

		(1) Never	(2)	(3)	(4)	(5) Always
C1.	Upset	1	2	3	4	5
C	Hostile	1	2	3	4	5

2.					
C3. Alert	1	2	3	4	5
C4. Ashamed	1	2	3	4	5
C5. Inspired	1	2	3	4	5
C6. Nervous	1	2	3	4	5
C7. Determined	1	2	3	4	5
C8. Attentive	1	2	3	4	5
C9. Afraid	1	2	3	4	5
C10. Active	1	2	3	4	5

I'm now going to ask you some demographic and general health questions.

D1. What is your age? ***(READ LIST)***
1 [ ] 65 to less than 70 years old
2 [ ] 70 to less than 75 years old
3[ ] 75 to less than 80 years old
4[ ] 80 to less than 85 years old
5[ ] 85 to less than 90 years old
6[ ] 90 to less than 95 years old
7[ ] 95 years old and older
(Refused)

D2. What is your marital status? ***(READ LIST)***
1[ ] Married 2[ ] Single 3[ ] Separated 4[ ] Live with Partner 5[ ] Widowed

D3. What is the highest level of education you have completed?
1[ ] Completed Bachelor degree or more
2[ ] Completed Associate degree or diploma
3[ ] Completed high school
4[ ] Some education
5[ ] Other (Please describe – RECORD VERBATIM)
(Refused)

D4. What is your ethnic group? (choose all that apply) ***(READ LIST)***

1[ ] White
2[ ] Black
3[ ] First Nations
4[ ] Inuit
5[ ] Metis
6[ ] Asian
7[ ] Other (Please describe – RECORD VERBATIM)
(Refused)

D5. Which culture do you identify with most? ***(READ LIST)***

1[ ] European
2[ ] Indigenous
3[ ] Asian
4[ ] African
5[ ] Latino
6[ ] Other (Please describe – RECORD VERBATIM)
(Refused)

D6. Which religion(s) do you practice? (choose all that apply) ***(READ LIST)***

1[ ] Christianity
2[ ] Judaism
3[ ] Islam
4[ ] Indigenous traditional religion
5[ ] Hinduism
5[ ] Buddhism
6[ ] Confucianism, or pray to Heaven or ancestors
7[ ] Taoism, or pray to the Gods of Chinese folklore
8[ ] I-Guann Daw
9[ ] Fallon Gung
10[ ] Atheism/Agnosticsm
11[ ] Other. Please describe. ______________________
(Refused)

D7. Are you an immigrant?

1[ ] Yes ***CONTINUE***
2[ ] No ***SKIP TO D8***
(Refused) ***SKIP TO D8***

D7A. How long have you been in Canada?

1. ______ (IF <1 PUT DECIMAL)
2. (Refused)

D7B. How long have you been in Saskatchewan?

1. ______ (IF <1 PUT DECIMAL)
2. (Refused)

D8. Is English your first language?
1[ ] Yes *SKIP TO D9*
2[ ] No *CONTINUE*
(Refused) *SKIP TO D9*

D8A. What is your first language?
1. (ENTERED RESPONSE)
2. (Refused)

D9. Do you have any extended health insurance?
1[ ] Yes
2[ ] No
(Don't Know)
(Refused)

D10. Are you able to access the services provided by your health benefit or insurance when you have a need? ***(READ LIST)***
1[ ] Yes for all type of services
2[ ] Yes for some types of services
3[ ] No. Please tell me why._________________
(Don't Know)
(Refused)

D11. Do you have any of the following six common chronic conditions? ***(READ LIST)***
1[ ] Asthma
2[ ] Chronic Obstructive Pulmonary Disease
3[ ] Coronary Artery Disease
4[ ] Depression
5[ ] Diabetes
6[ ] Heart Failure
7[ ] I do not have any of the six common chronic conditions.
(Refused)

D11A. In addition to those 6 chronic conditions, do you have any other chronic conditions? What are they? ***(RECORD RESPONSE VERBATIM)***
1. (ENTERED RESPONSE)
2. (Do not have other chronic conditions)
3. (Refused)

D12. Have you ever been a patient in an emergency room in the past three months?
1[ ] Yes (how many times?) ______ ***CONTINUE***
2[ ] No. ***SKP TO D13***
(Refused) ***SKP TO D13***

D12A. Did any of these emergency room visits lead to a hospitalization?
1[ ] Yes (How many of your emergency room visits led to a hospitalization?) ______
2[ ] No.
(Don't Know)
(Refused)

D13. Have you been hospitalized in the past three months?
1[ ] Yes (how many times?) ______ ***CONTINUE***
2[ ] No. ***SKP TO D14***
(Refused) ***SKP TO D14***

D13A. How many of your hospitalizations were for elective procedures or treatment? ________
1. (ENTERED RESPONSE)
2. (Don't Know)
3. (Refused)

D14. What is your most recent blood pressure reading? ____ / ______
1. (ENTERED RESPONSE)
2. (Don't Know)
3. (Refused)

D15. What is your most recent height?
1. Feet:
2. Inches:
3. Centimeters:
4. (Don't Know)
5. (Refused)

D16. What is your most recent weight?
1. Pounds:
2. Kilograms:
3. (Don't Know)
4. (Refused)

D17. In the past three months, were you able to meet your health care needs?
1[ ] Yes ***SKIP TO D18***
2[ ] No. ***CONTINUE***
(Refused) ***SKIP TO D18***

D17A. Can you describe the challenges you have encountered?
(RECORD RESPONSE VERBATIM)
1. (ENTERED RESPONSE)
2. (Don't Know)
3. (Refused)

D18. In the past three months, were you satisfied with your overall quality of life?
1[ ] Yes 2[ ] No
(Don't Know)
(Refused)

D19. In the past three months, were you satisfied with your overall physical health?
1[ ] Yes 2[ ] No
(Don't Know)
(Refused)

D20. In the past three months, were you satisfied with your overall mental health?
1[ ] Yes 2[ ] No
(Don't Know)
(Refused)

D21. In the past three months, were you satisfied with your overall emotional wellbeing?
1[ ] Yes 2[ ] No
(Don't Know)
(Refused)

D22. In the past three months, were you satisfied with your overall spiritual wellbeing?
1[ ] Yes 2[ ] No
(Don't Know)
(Refused)

D23. Do you have transportation when needed? 1[ ] Yes 2[ ] No
(Don't Know)
(Refused)

D24. What suggestions can you make to engage someone in their health and health care?
(RECORD RESPONSE VERBATIM)
1. (ENTERED RESPONSE)
2. (Don't Know)
3. (Refused)

I1. Are you willing to take part in future research activities such as focus groups or interviews on these topics?
1. Yes ***CONTINUE***
2. No ***SKIP TO END***
3. (Refused) ***SKIP TO END***

I2. Could we please have your name, phone number, and email address?
1. Name
2. Phone number
3. Email address
4. (Refused)

END. Thank you for your information and time. If you have any questions or concerns, you may contact the University of Saskatchewan Research Ethics Office at ethics.office@usask.ca or 306-966-2975. If you wish to follow-up with the study findings, you may contact the researcher, HM Tzeng at 306-966-5568. She can send you a summary of the outcomes of the study should you be interested.
